# Drug sensitivity of clinical isolates of *Mycobacterium tuberculosis* and its association with bacterial genotype in the Somali region, Eastern Ethiopia

**DOI:** 10.3389/fpubh.2022.942618

**Published:** 2022-08-17

**Authors:** Getnet Worku, Balako Gumi, Musse Girma, Binyam Mohammedbirhan, Getu Diriba, Getachew Seid, Melak Getu, Misikir Amare, Waganeh Sinshaw, Wondimu Ashagre, Rea Tschopp, Lauren Carruth, Gobena Ameni

**Affiliations:** ^1^College of Medicine and Health Sciences, Jigjiga University, Jigjiga, Ethiopia; ^2^Animal Health and Zoonotic Unit, Aklilu Lemma Institute of Pathobiology, Addis Ababa University, Addis Ababa, Ethiopia; ^3^Ethiopian Public Health Institute, National Tuberculosis Reference Laboratory, Addis Ababa, Ethiopia; ^4^One-Health Unit, Armauer Hansen Research Institute, Addis Ababa, Ethiopia; ^5^Department of Epidemiology and Public Health, Swiss Tropical and Public Health Institute, Basel, Switzerland; ^6^School of International Service, American University, Washington DC, DC, United States; ^7^Department of Veterinary Medicine, College of Agriculture and Veterinary Medicine, United Arab Emirates University, Al Ain, United Arab Emirates

**Keywords:** bacterial genotype, drug sensitivity, mycobacterium tuberculosis, performance of Xpert, Somali region of Ethiopia

## Abstract

**Background:**

Drug resistance is becoming a major bottleneck for tuberculosis (TB) control programs in countries with high TB burdens. Although several studies were conducted on the drug sensitivity of *Mycobacterium tuberculosis* (*M. tuberculosis*) in central Ethiopia, there is a lack of data on the drug sensitivity of *M. tuberculosis* in the peripheral regions of the country including in the Somali region. Therefore, the objective of this study was to evaluate the drug sensitivity of *M. tuberculosis* and its association with bacterial genotype and evaluate the performance of Xpert MTB/RIF (Xpert) in detecting resistance to rifampicin (RIF).

**Methods:**

A total of 302 *M. tuberculosis* were tested using the BD BACTEC-Mycobacteria Growth Indicator Tube 960 (MGIT 960) system for their drug sensitivity to the first-line anti-TB drugs. Besides, the drug sensitivity of 10 multidrug-resistant (MDR) *M. tuberculosis* isolates was evaluated for the second-line anti-TB drugs. Additionally, 177 of the 302 isolates were tested for genotypic drug resistance using Xpert. Chi-square and Fisher's exact tests were used for the evaluation of the association between variables and drug sensitivity.

**Results:**

The overall prevalence of resistance to at least one drug was 11.6% (95% CI: 7.9–15.2%), while the prevalence of MDR was 3.3% (95% CI: 1.3–5.3%). Two of the 10 MDR isolates were resistant to capreomycin. The spoligotype Shared International Type (SIT) 149 was significantly associated with either monoresistance or MDR (*p* < 0.05). Of the 177 isolates tested by Xpert, 6.2% (11/177) were RIF-resistant. Discordant between Xpert and MGIT 960 was observed in one isolate and linked with probe-binding delay (ΔCT max = 5.8). The sensitivity and specificity of the Xpert assay were 100 and 99.4%, respectively, while its positive and negative predictive values were 90.9 and 100%, respectively.

**Conclusion:**

The magnitude of MDR *M. tuberculosis* in the Somali region of Ethiopia was higher than the national prevalence of MDR-TB warranting the strengthening of the TB control program in the Somali region. Besides, drug resistance was associated with SIT 149 spoligotype (genotype). The Xpert assay was observed to have high sensitivity and specificity in detecting RIF-resistant *M. tuberculosis*, which is encouraging for its application widely.

## Introduction

Tuberculosis (TB) is a disease caused by *Mycobacterium tuberculosis* complex (MTBC) and affects all parts of the body although it primarily affects the lungs. TB is a communicable disease that is a major cause of illness and one of the leading causes of death worldwide; it used to cause the largest death from a single infectious agent until the coronavirus 2019 (COVID-19) pandemic. According to the latest report of WHO, there were 9.9 million cases and 1.5 million deaths due to TB in 2020 (1).

Tuberculosis is both curable and preventable, and as such a 6-month anti-TB drug regimen can successfully treat 85% of patients with TB (1). Drug resistance to anti-TB drugs emerged shortly after the introduction of the first drug streptomycin (STM) in clinical use (2). In recent years, resistance to isoniazid (INH) and rifampicin (RIF), the two most effective first-line treatments, is of particular concern; resistance to both drugs is defined as multidrug-resistant (MDR)-TB. MDR-TB and RIF-resistant TB (RR-TB) both require second-line treatment. Extensively drug-resistant TB (XDR-TB) is defined as MDR-TB strains, which are resistant to any fluoroquinolones and at least one additional Group A drug [levofloxacin (LEV), moxifloxacin, bedaquiline, and linezolid (LZD)] (3). Pre-extensively drug-resistant TB (Pre-XDR TB) is the MDR-TB strain that is resistant to any fluoroquinolones (3). In 2020, WHO estimated that of the 132,222 cases of MDR-TB, 25,681 were either XDR or pre-XDR-TB (1). The proportion of patients with MDR who were diagnosed with TB for the first time was around 3–4%, while for those who had previously been treated for TB was around 18–21% (1).

Although TB can be treated, detecting the disease is not always easy, and drug resistance is becoming more common (1). The development of rapid and accurate TB identification methods, particularly for smear-negative TB and MDR-TB, is critical for improving cure rates, reducing drug resistance and treatment failure, and preventing TB transmission (4). The nucleic acid amplification technique (NAAT) is now widely available to detect MTBC and drug resistance mutations from culture isolate or direct patient specimens. In 2010, the WHO endorsed the use of the GeneXpert platform Xpert MTB/RIF (Xpert) (Cepheid, Sunnyvale, CA, United States) assay, a modern, rapid, automated, cartridge-based NAAT that can simultaneously identify TB and RIF resistance, which is a surrogate marker for MDR-TB in resource-limited settings where phenotypic drug sensitivity tests (DSTs) are available only in reference laboratories (5, 6).

Genotyping techniques have greatly improved our understanding of TB epidemiology and have been used to guide disease control efforts. Based on molecular epidemiology technologies, some studies have found that drug resistance in *M. tuberculosis* is associated with a particular genotype (7). Several studies suggested that *M. tuberculosis* Beijing lineage may be associated with drug-resistant TB compared with the strain of other lineages (8–11). It is also been speculated that this association might be circumstantial since Beijing strains are more prevalent in places of the world where MDR-TB has emerged as a result of inadequate TB treatment. Furthermore, if Beijing strains spread more quickly than other lineages, it appears like they have more easily acquired mutations in MDR-TB hotspots (12).

In Ethiopia, TB remains one of the leading public health problems claiming the lives of thousands of Ethiopians every year. Ethiopia is among the 30 high TB burden and 30 high TB/HIV burden countries in the world. In the WHO global report from 2021, Ethiopia has been removed from the list of countries with a high MDR-TB burden (1). The first study performed on resistance to anti-TB drugs was in 1981 in Addis Ababa. In that study, there was no MDR-TB, while the first MDR-TB in Ethiopia was reported in 1986 with a prevalence of 1.1% (13). The first national anti-TB drug resistance study was conducted in 2005; MDR-TB was detected in 1.6 % of the newly diagnosed and 11.8 % of previously treated TB cases (14). According to the 2020 WHO global report, Ethiopia was among the high MDR-TB burden countries, with an estimated incidence of MDR/RR TB in 0.71% of new cases and 12% of previously treated cases in 2019 (15). The majority of TB drug resistance studies were conducted in only a few areas of the country and did not include marginalized pastoral areas such as the Somali region. Nonetheless, the less developed health infrastructure in pastoralist communities and the poor compliance with treatment due to patients' nomadic lifestyles contribute to the occurrence of MDR-TB in the Somali region (16). Therefore, the objective of this study was to evaluate the drug sensitivity profiles of *M. tuberculosis* strains using Xpert and MGIT 960 and to assess the association between drug resistance and the genetics of *M. tuberculosis* in the Somali region, Ethiopia.

## Methodology

### Study area and setting

This study was carried out in Jigjiga City, the capital of the Somali region, Ethiopia, the second largest region in the Federal Democratic Republic of Ethiopia. More than 83% of the population is rural and leads a pastoral or agro-pastoral way of life, with livestock serving as their primary source of income (17). The Somali region has long been facing unrest and instability that has severely hampered the government's capacity to provide basic social services to rural communities (18).

Patients with TB were recruited from three health facilities including Abilelie Health Center, Karamara Regional Hospital, and Jigjiga University Shiek Hassan Yabare Referral Hospital. These facilities were chosen as they represent the region's largest centers for TB diagnosis and treatment. Basic TB diagnostic facilities, such as smear microscopy, X-ray, and Xpert, were available in these facilities; however, only smear microscopy was available in the health center, and pathology laboratory service was only available at Jigjiga University Shiek Hassan Yabare Referral Hospital.

### Study design and study subjects

A health facility-based cross-sectional study was conducted on 336 patients with pulmonary and 156 patients with extrapulmonary TB visiting three selected health institutions in Jigjiga town between October 2018 and December 2019. The study subjects were bacteriologically confirmed patients with pulmonary TB and clinically suspected patients with extrapulmonary TB aged ≥15 years. Each patient provided informed consent to be included in this study. Patients who were unable to produce sputum and were less than 15 years were excluded from this study. Sputum and fine needle aspirates were collected and cultured from all patients with pulmonary TB and extrapulmonary TB, respectively. Culture positivity was confirmed in 323 samples of which 302 were successfully tested for drug sensitivity. Additionally, 177 of the 302 isolates were tested by Xpert, as part of a routine diagnosis for TB and RIF sensitivity test. In connection, the performance of Xpert in detecting RIF resistance was evaluated using MGIT 960 system as the gold standard.

### Sample collection and processing

Sputum samples were collected from each study participant by trained laboratory personnel, as recommended by WHO (19), and acid-fast bacilli or Xpert positive was stored in the refrigerator at −20°C at the sample collection site. Fine needle aspirates (FNAs) were collected and examined for cytology by an experienced pathologist under an aseptic procedure. The cytological diagnosis was performed on the first few drops of the aspirates, and the rest was stored in the refrigerator at −20°C at the sample collection site. All sputum specimens and FNA were transported in a packed ice box to the TB laboratory at the Aklilu Lemma Institute of Pathobiology (ALIPB), Addis Ababa University (AAU) at +4°C and stored at −80°C until further processing for culture. A structured questionnaire was used to collect patient variables including socio-demographic factors. Questionnaires were filled by trained nurses and laboratory technologists at each health institute.

### Xpert MTB/RIF

Xpert was performed at Karamara Hospital according to the manufacturer's instructions. A volume of 3–4 ml of sputum was mixed with sample reagent buffer (included in the kit) in 1:2 ratio, the tubes were vigorously agitated two times, and the tubes were left for 15 min at room temperature. The inactivated mixture was then transferred to the test cartridge with a sterile disposable pipette. The GeneXpert assay instrument was loaded with cartridges and generated results in <2 h. The trained laboratory personnel operated the Xpert modules and cartridges, which included specimen processing, invalid results handling, and standard protocol interpretation (5, 20).

The Xpert assay amplifies the 81-base pair portion of the *rpoB* (RNA polymerase enzyme β-subunit gene known as Rifampicin Resistance Determining Region (RRDR) and uses five probes (labeled A–E) to detect amplification. To signal “MTB detection,” at least two of five probes must be positive within a certain cycle threshold (Ct) window. Xpert assay additionally signals RR when probe dropout (no hybridization) or delayed Ct (ΔCt max is >4.0) of one to three probes occurs. The ΔCt max was calculated using the difference between the earliest (first) and latest (last) Ct across the five probes (21).

### Culture and identification

All samples were processed and cultured at ALIPB, AAU according to the Petroff technique (22). The specimens were decontaminated using an equal amount of 4% NaOH stock solution and sample and centrifuged at 3,000 rpm for 15 min. Then, the supernatant was discarded, and the sediment was neutralized with 2N HCl and finally inoculated onto two Löwenstein–Jensen (LJ) egg slant medium containing 0.6% sodium pyruvate and the other 0.75 % glycerol. The slants were incubated for at least 8 weeks with weekly observation for the presence of mycobacterial colonies. *M. tuberculosis* was identified from other members of the MTBC species using region of difference (RD)-9 polymerase chain reactions (PCR) from heat-killed cells (23). Spoligotyping was performed to characterize all isolates, as described previously by Kamerbeek (24). In brief, the primers DRa (biotinylated at the 5'end) and DRb were used to amplify the direct repeat (DR) region using the PCR machine. The amplified products were hybridized with a series of 43 immobilized oligonucleotides covalently bound to a membrane, each of which corresponds to a distinct spacer DNA sequence found within the DR locus of MTBC. Hybridized DNA was identified using the enhanced chemiluminescence technique after incubation with streptavidin-peroxidase, which binds to the biotin label on the PCR product, and exposure to X-ray film in accordance with the manufacturer's instructions. The presence and absence of spacers were represented on the X-ray film by squares that were black or white, respectively. *M. tuberculosis* H37Rv and *Mycobacterium bovis* strains were used as positive controls, while distilled water was used as the negative control. The spoligotype patterns were converted into binary and octal formats and entered into the online spoligotype database to determine SIT number, and the results were compared with already existing designations in the international spoligotyping database (http://www.pasteur-guadeloupe.fr:8081/SITVIT2/).

### Phenotypic drug sensitivity tests

A drug sensitivity test was performed at National Tuberculosis Reference Laboratory, Ethiopian Public Health Institute (EPHI). The BD BACTEC-MGIT 960 system was used to test four first-line drugs using the SIRE kit: STM, INH, RIF, and ethambutol (EMB). All MDR-TB isolates were tested to second-line DST using MGIT 960 systems. The primary isolates were diluted to 1:100 before DST was conducted, and DST was performed within 1–5 days of a positive growth result. The final drug concentrations for first-line drugs STM, INH, RIF, and EMB were 1.0, 0.1, 1, and 5.0 μg/ml, respectively. The final drug concentration for second-line drugs were amikacin (AMK) 1.0 μg/ml, kanamycin (KAN) 2.5 μg/ml, capreomycin (CAP) 2.5 μg/ml, ofloxacin (OFX) 2 μg/ml, LEV 1.5 μg/ml, clofazimine (CFZ) 1 μg/ml, and LZD 1 μg/ml. A growth control tube was used for each isolate, containing a growth supplement but no drug. The MGIT 960 system's software algorithm determined the relative growth ratio between the drug-containing tube and the drug-free growth control tube. Then, the instrument automatically determines the final interpretation and susceptibility results. If 1% or more of the test population grows in the presence of the critical concentration of the drug, the isolate is considered resistant (25).

### Line probe assay

Line probe assay (LPA) (Genotype MTBDR*plus*) was performed to further investigate the discordant result between Xpert and MGIT 960 according to the manufacturer's instructions in the EPHI laboratory (26).

### Data analysis

All components of the data were entered and analyzed using SPSS 20 computer software. Descriptive statistics were used to depict the socio-demographic features. The relationship between drug resistance and bacterial and host-related factors was analyzed using Pearson's χ^2^ test or Fisher's exact test as appropriate. MedCalc Statistical Software version 20.106 was used to calculate the test performance of Xpert. Statistical significance was determined at *p* < 0.05.

## Results

### Magnitude of drug resistance and its association with demographic/clinical characteristics of the patients

The prevalence of drug resistance to at least one first-line anti-TB drug was 11.6% (95% CI: 7.9–15.2), and MDR was observed in 3.3% (95% CI: 1.7–5.6) of the isolates. The results of sensitivity tests for first-line anti-TB drugs are shown in Table 1. Of the 10 MDR *M. tuberculosis* isolates tested for the second-line DST, only two isolates were found to be resistant to CAP. Thus, according to the new WHO publication (3), none of the 10 MDR isolates was either pre-XDR TB or XDR TB.

**Table 1:** Drug resistance profiles of new and previously treated patients with tuberculosis (TB) in the Somali Region, Ethiopia (*n* = 302).

	**Previously treated cases, *n* = 12**	**New cases, *n* = 290**	**Total,** ***n* = 302**
First line drugs	*n* (%)	*n* (%)	*n* (%)
Resistant to at least one drug	6 (50)	29 (10)	35 (11.6)
**Resistant to any of the drugs**			
STM	2 (16.7)	21 (7.2)	23 (7.6)
INH	4 (33.3)	12 (4.1)	16 (5.3)
RIF	2 (16.7)	8 (2.8)	10 (3.3)
EMB	0	0	0
**Poly resistance**			
STM + INH	0	2 (0.7)	2 (0.7)
RIF + INH	2 (16.7)	6 (2.1)	8 (2.6)
STM + INH + RIF	0	2 (0.7)	2 (0.7)
All MDR	2 (14.3)	8 (2.8)	10 (3.3)
**Monoresistance**			
STM	2 (16.7)	17 (5.9)	19 (6.3)
INH	2 (16.7)	2 (0.7)	4 (1.3)

STM, streptomycin; INH, isoniazid; RIF, rifampicin; EMB, ethambutol; MDR, multidrug-resistant.

A total of 323 MTBC were isolated from 249 sputum 249 and 74 FNA samples during the study period. But following sub-culturing on MGIT for DST, 2.8% (9/323) of the isolate lost viability while 3.7% (12/323) of the isolates were contaminated. Thus, these two groups were excluded from the final analysis. Therefore, 302 isolates (232 from pulmonary TB cases and 70 from extrapulmonary TB cases) were tested for drug sensitivity. The 302 patients who were the source of the isolates comprised 64.9% (196/302 male, 60.3% (182/302) urban residents, and 76.8% (232/302) pulmonary TB patients. Among extrapulmonary specimens 95.7% (67/70) were collected from lymph nodes [with cervical lymph node 88.6% (62/70)], 2.9% (2/70) from skin lesions, and 1.4% (1/70) from breast abscess. The median age of the patients was 28 years (range: 15 to 80 years) and 81.8% (247/302) were in the age group of 15–44 years.

The socio-demographic characteristics of the study participants are summarized in Table 2. Previous anti-TB drug treatment was associated with resistance to one or more drugs (χ^2^ = 17.9, *p* < 0.05), and MDR (χ^2^ = 6.9, *p* = 0.055). The proportion of drug-resistant strains for one or more drugs was similar in extrapulmonary TB (12.9%) and pulmonary TB (11.2%) (χ^2^ = 0.14, *p* = 0.705), and no MDR-TB strains were isolated from extrapulmonary TB (χ^2^ = 3.12, *p* = 0.124). Table 2 shows the relationship between drug sensitivity and demographic and clinical features of study participants.

**Table 2:** Demographic characteristics and their relationship with drug resistance in patients with TB in Somali region, Ethiopia (*n* = 302).

**Characteristics**		**Resistant to one or more drugs *n* (%)**	**95% CI**	**χ^2^ test**	***p*-value**	**MDR *n* (%)**	**95% CI**	**χ^2^ test**	***p*-value**
Sex	Female	15 (14.2)	7.5 −21.7	1.06	0.306	4 (3.8)	0.9 −7.5	0.109	0.745
	Male	20 (10.2)	6.1−14.3			6 (3.1)	1–5.		
Residence	Rural	16 (13.3)	7.5–20	0.591	0.442	2 (1.7)	0–4.2	1.682	0.325
	Urban	19 (10.4)	6–15.4			8 (4.4)	1.6–7.7		
Contact History	Yes	7 (11.9)	5.1–22	0.005	0.941	4 (6.8)	1.7–13.6	2.755	0.109
	No	28 (11.5)	7.8–15.6			6 (2.5)	0.8–4.5		
Type of TB	Extra-pulmonary TB	9 (12.9)	5.7–21.4	0.143	0.705	0	0	3.121	0.124
	pulmonary TB	26 (11.2)	7.3–15.5			10 (4.3)	1.7–6.9		
History of Treatment	Previously Treated	6 (50)	25–75	17.9	0.001*	2 (16.7)	0–41.7	6.962	0.055
	New	29 (10)	6.6–13.8			8 (2.8)	1–4.8		
Age Group	15–24	12 (10.2)	5.1–16.1	2.488	0.477	4 (3.4)	0.8–6.8	0.674	0.914
	25–34	12 (15)	7.5–23.8			3 (3.8)	0–8.8		
	35–44	7 (14.3)	4.1–24.5			2 (4.1)	0–10.2		
	45	4 (7.3)	1.8–14.5			1 (1.8)	0–5.5		

CI, confidence interval; MDR, multidrug-resistant.

*Statistically significant.

### Association between drug resistance and bacterial genotype

The associations between drug resistances with isolated bacterial genotypes are presented in Table 3. The SIT 149 (T3-ETH) spoligotype was significantly associated with resistance to one or more drugs (χ^2^ = 13.9, *p* < 0.05) and MDR (χ^2^ = 12.4, *p* < 0.05) when compared with the other spoligotypes. The 10 MDR isolates were grouped as SIT 149 (T3-ETH), SIT 37 (T3), and SIT 21 (CAS1-Kili), which accounted for 70% (7/10), 20% (2/10), and 10% (1/10) of the isolates, respectively. One of the MDR isolates was identified during household contact investigation according to the Ethiopian TB guidelines (1, 27) and had the same spoligotype pattern as the index case SIT 149 (T3-ETH). Both clustered MDR SIT 37 isolates were resistant to the second-line anti-TB drug CAP.

**Table 3:** Genotype of *Mycobacterium tuberculosis* and its association with drug resistance in the Somali region, Ethiopia (*n* = 302).

**Characteristics**		**Resistant to one or more drug *n* (%)**	**95% CI**	**χ^2^ test**	***p*-value**	**MDR *n* (%)**	**95% CI**	**χ^2^ test**	***p*-value**
Dominant SIT	SIT 149	13 (24.1)	13–35.2			7 (13)	3.7–22.2		
	SIT 21	6 (14.6)	4.9–26.8	13.9	0.005*	1 (2.4)	0–7.3	12.42	0.005*
	SIT 26	2 (7.4)	0–18.5			0	0		
	SIT 53	4 (19)	4.8–38.1			0	0		
	Others	10 (6.3)	2.5–10.7			2 (1.3)	0–3.1		
Clustering	Clustered	32 (12.3)	8.8–16.1	0.845	0.443	10 (3.8)	1.5–6.5	1.625	0.368
	Unique	3 (7.3)	0–17.1			0	0		

SIT, Shared International Type; MDR, multidrug-resistant.

*statistically significant.

### Genotypic drug resistance detection by Xpert MTB/RIF

Xpert was performed on 177 isolates of the 232 isolates recovered from pulmonary TB cases as a part of routine diagnosis. RIF resistance was detected in 6.2% (11/177) of the isolates, while the remaining 93.8% (166/177) were RIF sensitive. RIF indeterminate results were not found in any of the isolates. RIF resistance mutations result in complete dropout (no hybridization) on probe E (codons 529–533) in nine isolates and complete dropout on probe D (codons 523–529) in one isolate. RIF discordant result occurred between Xpert and MGIT 960 in one isolate and was associated with probe-binding delay (Δ*C*_T_ max = 5.8). On further analysis using the LPA, RR mutation was detected. But it is not due to hybridization with the known mutant probes (MUT1, MUT2A, MUT2B, and MUT3) rather by the absence of hybridization with the wild-type probe 8 (WT8) (codons 530–533).

### Sensitivity and specificity of Xpert MTB/RIF for the detection of RIF resistance

Of the 177 isolates tested by Xpert assay, 6.2% (11/177) were RIF-resistant. Discordant between Xpert and MGIT 960 was observed in one isolate and linked with probe-binding delay (Δ*C*_T_ max = 5.8). The sensitivity and specificity of the Xpert assay were 100 and 99.4%, respectively, while its positive and negative predictive values were 90.9 and 100%, respectively (Table 4).

**Table 4:** Diagnostic performance of Xpert MTB/RIF for the detection of rifampicin resistance as compared with gold standard MGIT 960.

**Performance**	**Value**	**95% CI**
Sensitivity	100.0%	69.2–100
Specificity	99.4%	96.7–99.9
PPV	90.9%	58.6–98.6
NPV	100.0%	
Accuracy	99.4%	96.9–99.9

NPV, negative predictive value; PPV, positive predictive value; CI, confidence interval.

## Discussion

This study was conducted to evaluate the drug sensitivity profile of 302 *M. tuberculosis* isolated from the Somali region of eastern Ethiopia. Both phenotypic and genotypic methods were used in this study. This could be considered important as it is the first study that was conducted on the evaluation of drug resistance in *M. tuberculosis*, which is critical for successful TB treatment and future healthcare planning.

The prevalence of drug resistance recorded in new patients was comparable with the results of studies conducted in Gondar (28) and South Omo (29); however, it was relatively higher than the prevalence reported in northern Ethiopia (30). Higher prevalence values were also reported from southwestern Ethiopia (31), central Ethiopia (32), eastern Ethiopia (33), and south Ethiopia (34). The differences in the prevalence of drug resistance reported from the different regions of Ethiopia could be caused by different factors. It could be due to variations in the strength of the TB control programs at the study sites, treatment compliance of the patients, the economic status (nutritional status) of the patients, the immunological status of the patients, and variation in the virulence of the bacterial strain.

The prevalence of MDR in the new and previously treated patient was similar to the prevalence of MDR reported by studies conducted in northwest Ethiopia (35); but higher than the prevalence values reported from different parts of Ethiopia (29, 31, 32), although a very high prevalence (61.9%) of MDR was reported from Addis Ababa (36).

Regarding mono-resistance, STM mono-resistance was the most prevalent and is comparable with the results of previous studies in Ethiopia (33, 37), while some other studies reported found a lower percentage of STM mono-resistance (31, 38). Nonetheless, a relatively high prevalence of STM mono-resistance was reported using a large-scale Ethiopian community survey (39) and from the Amhara region (40). INH mono-resistance was lower than those reported by others in the country (31, 33, 36, 37). Mono-resistance to RIF and EMB was not identified in our study. A low prevalence of RIF and EMB resistance was observed previously in Ethiopia (30, 40–42). In contrast to the reports of the previous studies conducted in Ethiopia (36, 43), no XDR cases and pre-XDR were detected in this study. The possible reasons for the differences in either mono-resistance of MDR are indicated above and related to either patient factors, TB control program, and/or pathogen factors.

The SIT 149 that belongs to lineage four exhibited a high frequency of drug resistance, and the association between this spoligotype and drug resistance was significant. It is established that Beijing (lineage 2) is associated with MDR globally although the lineage is less frequent in Ethiopia (7, 44, 45). The observation of an association between SIT 149 and drug resistance was also observed by other studies (43, 46, 47). SIT 149 is the most frequently isolated spoligotype from different regions of Ethiopia. So, the observation association between SIT 149 and drug resistance could be due to its frequent occurrence in Ethiopia which can also be reflected in the drug-resistant isolates. Such observation was reported by a review that was published by Panwalkar et al. (48). It can also reflect the real association between bacterial genetics and drug resistance, which could be due to mutation in the genes of isolates with SIT 149 spoligotype that guarantees them drug resistance.

The prevalence of drug resistance was comparable between extrapulmonary TB and pulmonary TB in this study. But no MDR-TB was in the isolates of extrapulmonary TB in this study. So far, no extensive study was conducted on the comparison of drug resistance in pulmonary TB and extrapulmonary isolates in Ethiopia while studies from India (49) and Korea (50) indicated that MDR-TB was less common in patients with extrapulmonary TB than in patients with pulmonary TB.

The observation of seven isolates with SIT 149 spoligotype pattern and two isolates with SIT 37 spoligotype pattern means that the observation of clustered patterns could suggest recent transmission of MDR-TB in the study area. Substantiating this hypothesis, in this study, it was observed that one of the MDR isolates was isolated from the same household as the index case although the discriminating power of spoligotyping is generally low (51).

The results of culture-based phenotypic drug susceptibility testing procedures can take weeks to months. Molecular-based testing such as Xpert, in contrast, can provide results within 2 h. Therefore, many countries have incorporated Xpert into their routine TB diagnostic algorithms. Xpert is one of the most useful tests for detecting RIF resistance, which acts as a proxy for MDR-TB and predicts poor treatment outcomes. The assays detect mutations in the 81-base-pair *rpoB* gene, which is linked to RIF resistance in 95–98% of cases (5). Detecting RR by Xpert is a critical element of TB care for both the individual patient and the public health since it starts a sequence of events that includes further DST, contact investigation, and patient referral for potentially toxic and less effective MDR therapy (52). The Xpert has high sensitivity and specificity for identifying RR in patients with pulmonary TB. Similar results were reported by a meta-analysis, which reported a pooled sensitivity of 96% and a specificity of 98% (53).

In this study, 11 RIF-resistant isolates on Xpert were also resistant to INH on the MGIT 960 phenotypic test, indicating that the Xpert test is a useful test for detecting MDR-TB in resource-limited settings. However, one isolate was susceptible to RIF on MGIT 960 phenotypic test. This discordant (false positive) result was due to probe delay evident in this study (ΔCt = 5.8). To rule out the possibility that low bacterial load affected the initial Xpert assay results by delaying Probe E binding (54), we retested the Xpert assay from the MGIT test's growth control, and the results were similar. This isolate was further evaluated by LPA, and unknown RIF-resistance mutations were detected owing to the absence of WT8 hybridization rather than hybridization with known mutation probes. This false positive RIF-resistant molecular tests result might be due to a silent mutation rather than real resistance, which could explain the delayed probe E binding on Xpert and the absence of binding to known mutation probes on LPA (55). Alternatively, the unusual occurrence of false positive results might be attributed to the detection of RR strains by Xpert but not by the current reference standard and the phenotypic culture-based DST (MGIT 960) (4). In this study, the most prevalent mutation (9 out of 10) of the MDR isolates was found in the codons 529–533 (probe E), and the rest (1 out of 10) was detected in codons 523–529 (probe D). A similar observation was made earlier in Ethiopia (56). Furthermore, similar results were reported from Nigeria, Uganda, and Pakistan (57–59).

## Conclusion

The magnitude of MDR isolates of *M. tuberculosis* in the Somali region of Ethiopia was higher than the national prevalence of MDR-TB warranting the strengthening of the TB control program in the Somali region of Ethiopia. Particularly, drug resistance was common in isolates with the spoligotype SIT 149, which was widespread in Ethiopia. The Xpert assay was observed to have high sensitivity and specificity in detecting RIF-resistant *M. tuberculosis*, which is encouraging for its application widely.

## Data availability statement

The raw data supporting the conclusions of this article will be made available by the authors, without undue reservation.

## Ethics statement

The studies involving human participants were reviewed and approved by the Institutional Review Board (IRB) of the Aklilu Lemma Institute of Pathobiology, Addis Ababa University Ref No. ALIPB/IRB/002/2017/18. Permission was requested from Ethiopian Somali Regional State Health bureau and each study sites. The study was explained to the patients, and consent for participation was obtained prior to collecting the specimens. The patients/participants provided their written informed consent to participate in this study.

## Author contributions

GW contributed to designing this study, data collection, analysis, drafting of the manuscript, analysis of and interpretation of the result, and edition of the manuscript. BG supervised the study and edited the manuscript. MG, BM, GD, GS, MG, MA, WS, and WA contributed to the field data collection, culturing of samples, and drug sensitivity testing. RT and LC contributed to the edition of the manuscript. GA contributed to conceptualizing and designing this study, leading, and supervising. All authors contributed to this study and approved the submitted version.

## Funding

The research project received a small amount of financial support from the American University, USA. In addition, the research project obtained support in kinds such as reagents and research supplies from the Ethiopian Public Health Institute (EPHI), the Swiss Agency for Development and Cooperation (SDC), the Jigjiga University, the Armauer Hansen Research Institute (AHRI), and the Addis Ababa University.

## Conflict of interest

The authors declare that the research was conducted in the absence of any commercial or financial relationships that could be construed as a potential conflict of interest.

## Publisher's note

All claims expressed in this article are solely those of the authors and do not necessarily represent those of their affiliated organizations, or those of the publisher, the editors and the reviewers. Any product that may be evaluated in this article, or claim that may be made by its manufacturer, is not guaranteed or endorsed by the publisher.
